# Supplementary material for: Sensory receptor expansion and neural accommodation in butterfly color vision
Source: Sci Adv. 2026 Oct 7;12(41):eaei7570. doi: 10.1126/sciadv.aei7570 (PMC13644764; doi:10.1126/sciadv.aei7570)
Supplement: Supplementary file 1 [file sciadv.aei7570_sm.pdf]

Science Advances
AAAS

Supplementary Materials for

Sensory receptor expansion and neural accommodation in butterfly color vision

Ke Gao *et al.*

Corresponding author: Michael W. Perry, mwperry@ucsd.edu

Sci. Adv. **12**, eaei7570 (2026)
DOI: 10.1126/sciadv.aei7570

The PDF file includes:

Figs. S1 to S10
Tables S1 to S4
Legend for data S1
References

Other Supplementary Material for this manuscript includes the following:

Data S1

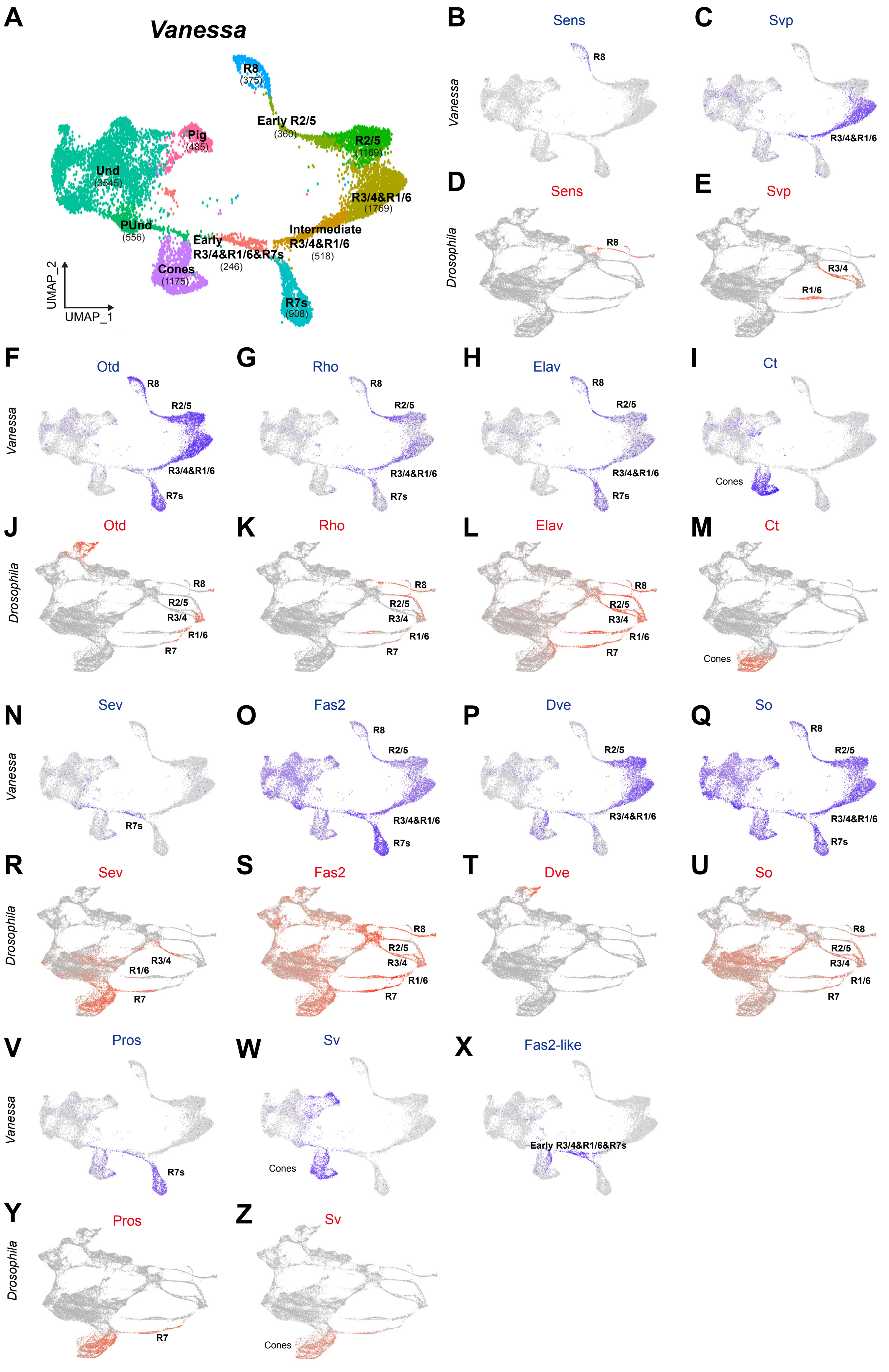
A
Vanessa
R8
(375)
Early R2/5
(360)
Pig
(485)
R2/5
(1169)
Und
(3545)
R3/4&R1/6
(1769)
PUnd
(556)
Intermediate
R3/4&R1/6
(518)
Early
R3/4&R1/6&R7s
(246)
Cones
(1175)
R7s
(908)
UMAP_2
UMAP_1
B Sens Vanessa R8
C Svp R3/4&R1/6
D Sens Drosophila R8
E Svp R3/4 R1/6
F Otd Vanessa R8 R2/5 R3/4&R1/6 R7s
G Rho R8 R2/5 R3/4&R1/6 R7s
H Elav R8 R2/5 R3/4&R1/6 R7s
I Ct Cones
J Otd Drosophila R8 R2/5 R3/4 R1/6 R7
K Rho R8 R2/5 R3/4 R1/6 R7
L Elav R8 R2/5 R3/4 R1/6 R7
M Ct Cones
N Sev Vanessa R7s
O Fas2 R8 R2/5 R3/4&R1/6 R7s
P Dve R2/5 R3/4&R1/6
Q So R8 R2/5 R3/4&R1/6 R7s
R Sev Drosophila R3/4 R1/6 R7
S Fas2 R8 R2/5 R3/4 R1/6 R7
T Dve
U So R8 R2/5 R3/4 R1/6 R7
V Pros Vanessa R7s
W Sv Cones
X Fas2-like Early R3/4&R1/6&R7s
Y Pros Drosophila R7
Z Sv Cones

Figure S1. FeaturePlots highlighting TF expression in the cell types of *Drosophila* and *Vanessa.*

(**A**) UMAP plot showing clusters with a total of 11,106 cells from the snRNAseq data of the butterfly P20 pupal retina. The number of cells in each cluster is shown in brackets. Early R3/4/1/6 + Intermediate R3/4/1/6 = 764, Early R2/5 = 360. (**B** to **Z**) FeaturePlots showing the expression of TFs in PRs of *Drosophila* and *Vanessa*. Und, undifferentiated; PUnd, posterior undifferentiated; Pig, pigment.

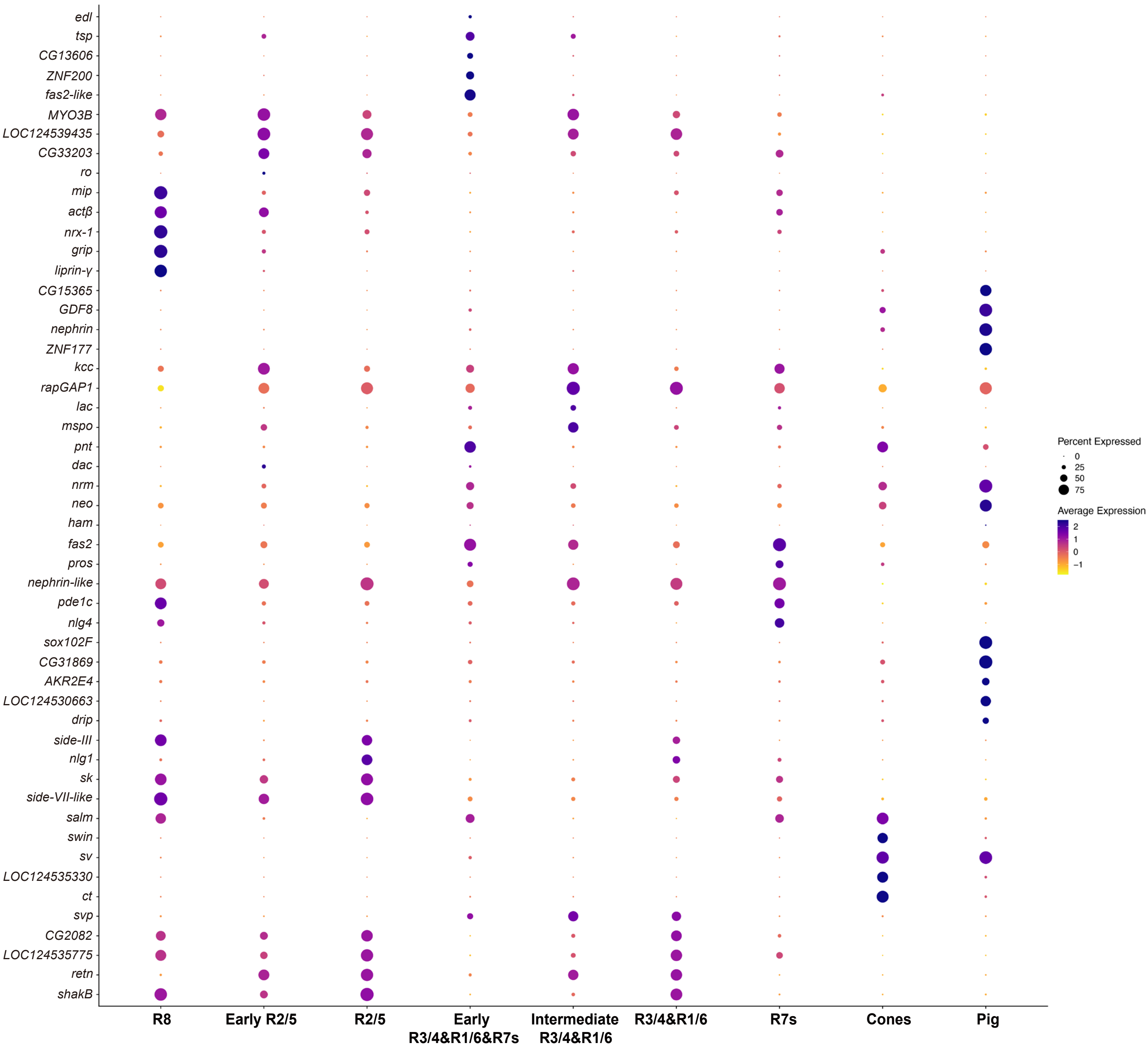

edl
tsp
CG13606
ZNF200
fas2-like
MYO3B
LOC124539435
CG33203
ro
mip
actβ
nrx-1
grip
liprin-γ
CG15365
GDF8
nephrin
ZNF177
kcc
rapGAP1
lac
mspo
pnt
dac
nrm
neo
ham
fas2
pros
nephrin-like
pde1c
nlg4
sox102F
CG31869
AKR2E4
LOC124530663
drip
side-III
nlg1
sk
side-VII-like
salm
swin
sv
LOC124535330
ct
svp
CG2082
LOC124535775
retn
shakB
R8
Early R2/5
R2/5
Early R3/4&R1/6&R7s
Intermediate R3/4&R1/6
R3/4&R1/6
R7s
Cones
Pig
Percent Expressed
0
25
50
75
Average Expression
2
1
0
−1

Figure S2. Marker gene expression within cell types. DotPlot showing the top 51 genes associated with each cell type cluster.

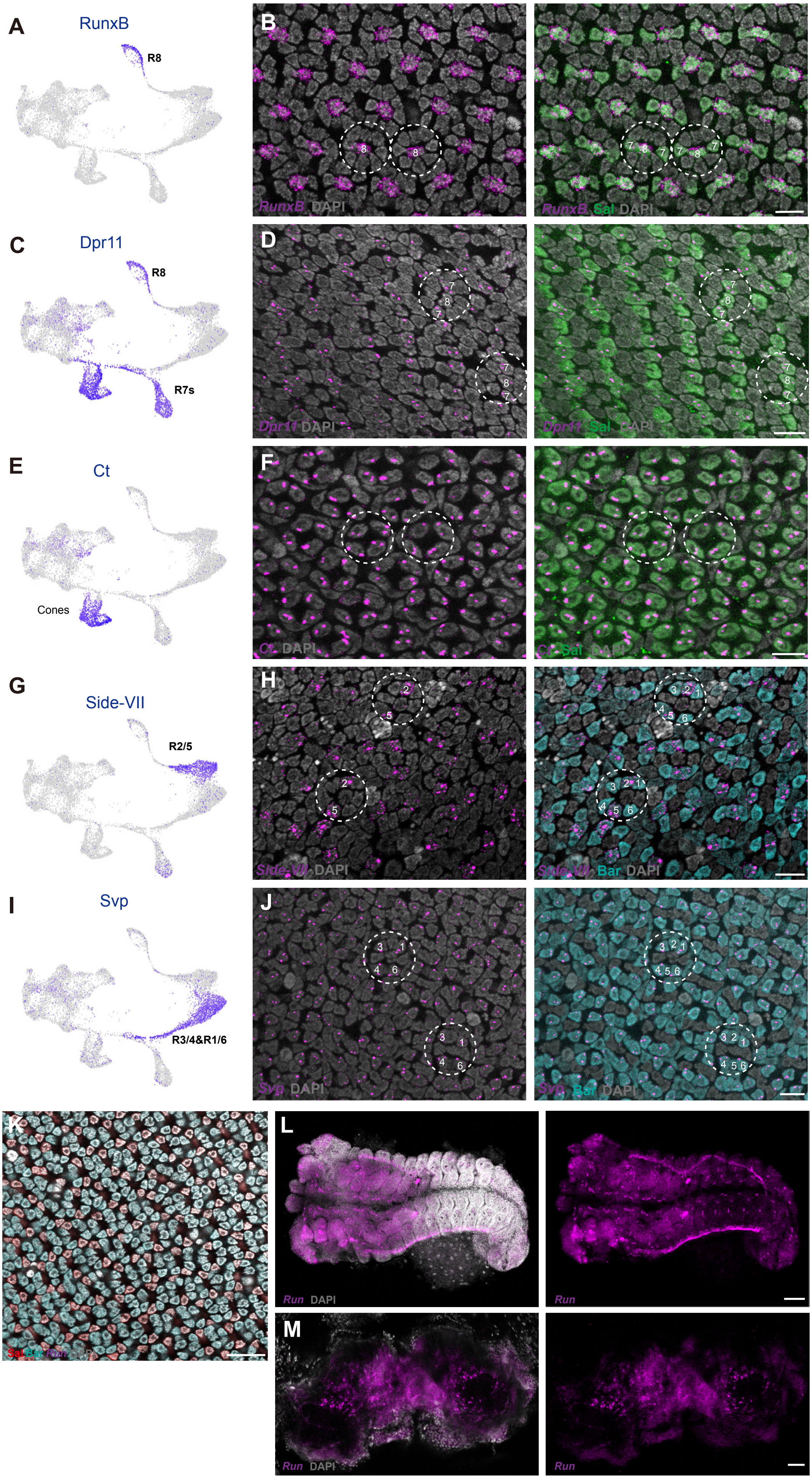
A
RunxB
R8
B
RunxB DAPI
RunxB Sal DAPI
C
Dpr11
R8
R7s
D
Dpr11 DAPI
Dpr11 Sal DAPI
E
Ct
Cones
F
Ct DAPI
Ct Sal DAPI
G
Side-VII
R2/5
H
Side-VII DAPI
Side-VII Bar DAPI
I
Svp
R3/4&R1/6
J
Svp DAPI
Svp Bar DAPI
K
L
Run DAPI
Run
M
Run DAPI
Run

Figure S3. Validation of cell type clusters using HCR *in situ* hybridization and antibody staining in *Vanessa.*

(**A** and **B**) RunxB is expressed in R8 cells. FeaturePlot showing *RunxB* expression in R8 cluster (A). HCR *in situ* hybridization combined with antibody staining shows *RunxB* mRNA expression (magenta) in R8, Sal (green) labels R8 and R7s. (**C** and **D**) Dpr11 is expressed in R8 and R7 cells. FeaturePlot showing *Dpr11* expression in R8 and R7s cluster (C). HCR *in situ* hybridization combined with antibody staining shows *Dpr11* mRNA expression (magenta) in R8 and R7s, Sal (green) labels R8 and R7s. (**E** and **F**) Ct is expressed in cone cells. FeaturePlot showing *Ct* expression in cone cells cluster (E). HCR *in situ* hybridization combined with antibody staining shows *Ct* mRNA expression (magenta) in cone cells, Sal (green) labels cone cells. (**G** and **H**) Side-VII is expressed in R2/5 cells. FeaturePlot showing *Side-VII* expression in R2/5 cells cluster (G). HCR *in situ* hybridization combined with antibody staining shows *Side-VII* mRNA expression (magenta) in R2/5, Bar labels outer PRs, R1-6 (cyan). (**I** and **J**) Svp is expressed in R3/4 and R1/6 cells. FeaturePlot showing *Svp* expression in R3/4&R1/6 cells cluster (I). HCR *in situ* hybridization combined with antibody staining shows *Svp* mRNA expression (magenta) in R3/4 and R1/6, Bar (cyan) labels outer PRs, R1-6. B to J, scale bar 10μm. (**K**) *Runt* mRNA expression is not detected by HCR *in situ* hybridization combined with antibody staining, only Sal (red) and Bar (cyan) expression is observed. Scale bar 20μm. (**L** and **M**) *Runt* mRNA expression (magenta) is observed in the embryo (L) and adult brain (M). L, scale bar 100μm. M, scale bar 50μm. All nuclei are labeled with DAPI (gray).

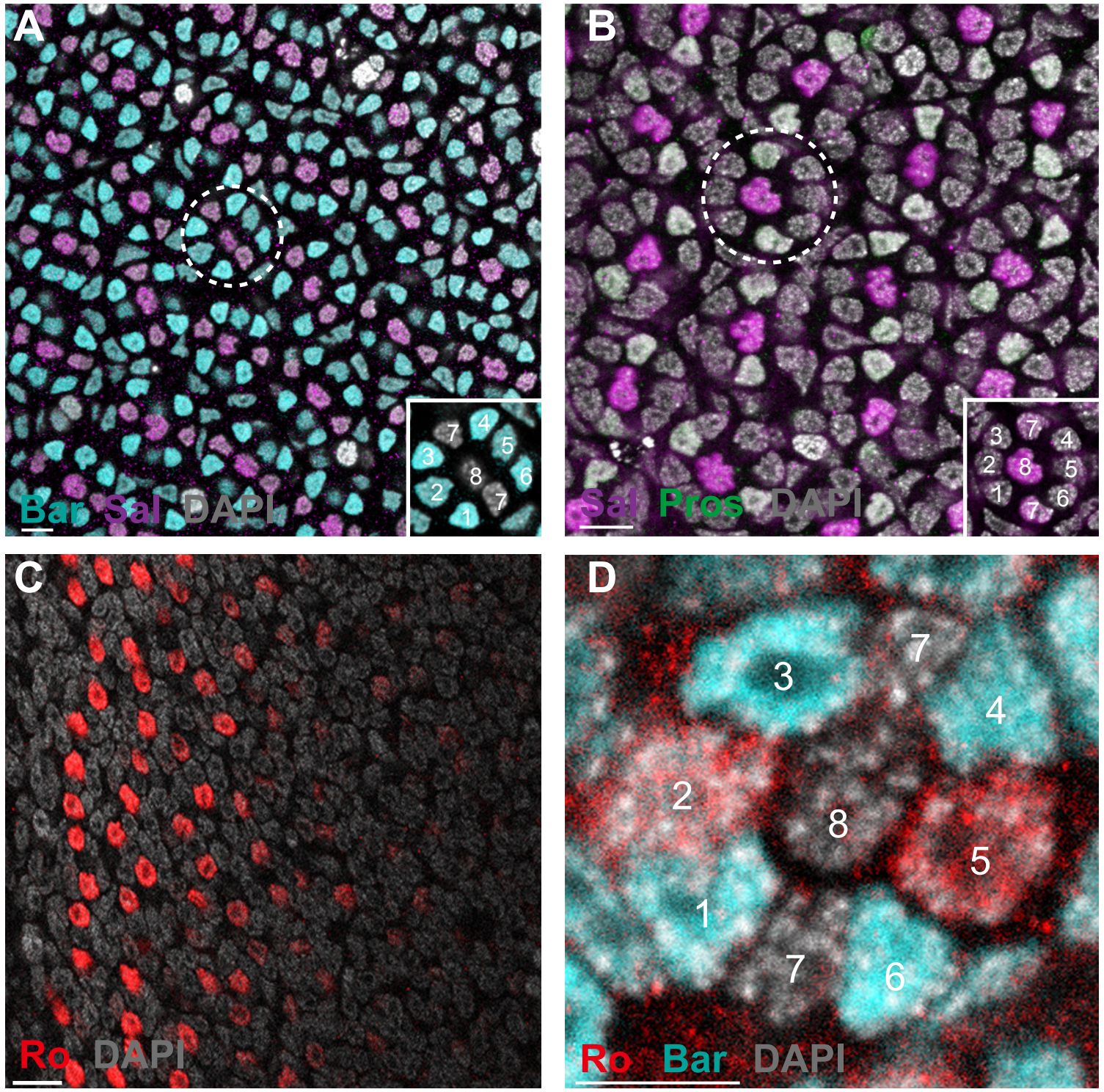
A
Bar Sal DAPI
7
4
5
3
8
6
2
7
1
B
Sal Pros DAPI
3
7
4
2
8
5
1
7
6
C
Ro DAPI
D
3
7
4
2
8
5
1
7
6
Ro Bar DAPI

Figure S4. Expression of four TFs in butterfly PRs.

(**A**) Antibody stains showing ommatidia in the butterfly *Vanessa*. Bar (cyan) is expressed in outer PRs, R1-6. An ommatidium with nine PRs is shown within a dashed circle (also see inset). Scale bar, 10μm. (**B**) Sal (magenta) is expressed in R8 and R7 PRs. Pros (green) is expressed in two R7s. The dashed circle highlights an ommatidium (also see inset). Scale bar, 10μm. (**C**) Ro (red) is transiently expressed near the morphogenetic furrow. Scale bar, 10μm. (**D**) An ommatidium near morphogenetic furrow showing Ro expression in R2/5, and Bar expression in R3/4 and R1/6. Scale bar, 5μm. All nuclei are labeled with DAPI (gray).

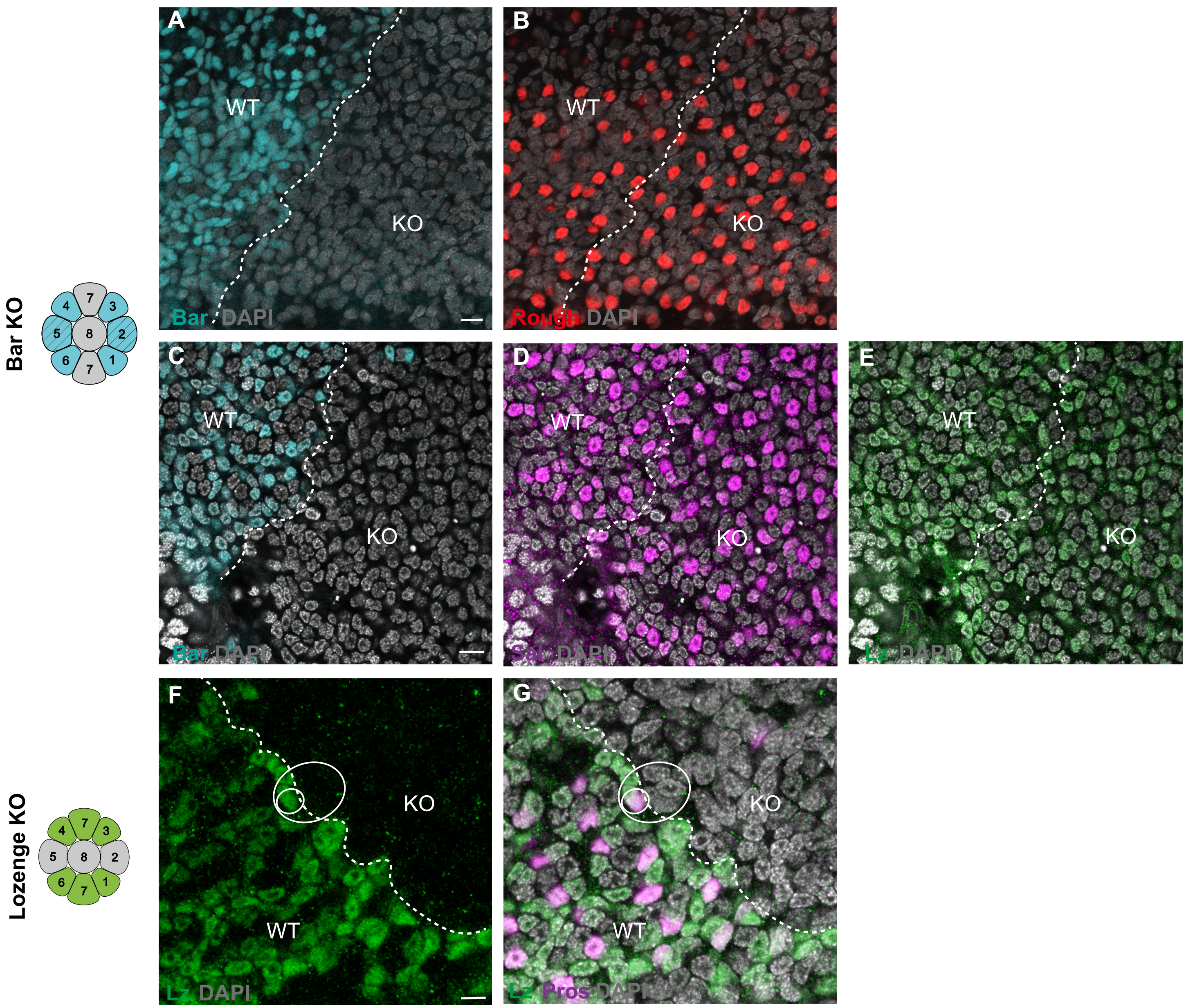

Bar KO
Lozenge KO
A
WT
KO
Bar DAPI
B
WT
KO
Rough DAPI
C
WT
KO
Bar DAPI
D
WT
KO
Sal DAPI
E
WT
KO
Lz DAPI
F
KO
WT
Lz DAPI
G
KO
WT
Lz Pros DAPI

Figure S5. The effects of Bar and Lozenge loss-of-function in the butterfly *Vanessa* retina.

(**A** to **E**) CRISPR-induced mosaic knockout of *Bar* does not affect the expression of Ro (red, B), Sal (magenta, D) or Lz (green, E) in the pupal retina of *Vanessa*. Bar (cyan, A and C) is lost in KO regions compared to the WT region. The boundary between WT and Bar KO regions is indicated by a white dashed line. Scale bar, 5µm. (**F** and **G**) CRISPR-induced mosaic knockout of *lz* results in the loss of R7s in the pupal retina of *Vanessa*. The solid white circle highlights an ommatidium near the clone boundary that has lost one R7 (the remaining R7 is stained with Lz (green, F) and Pros (magenta, G) on WT side) indicating that Lz is directly required in R7 recruitment. All nuclei are labeled with DAPI (gray). Scale bar, 10µm.

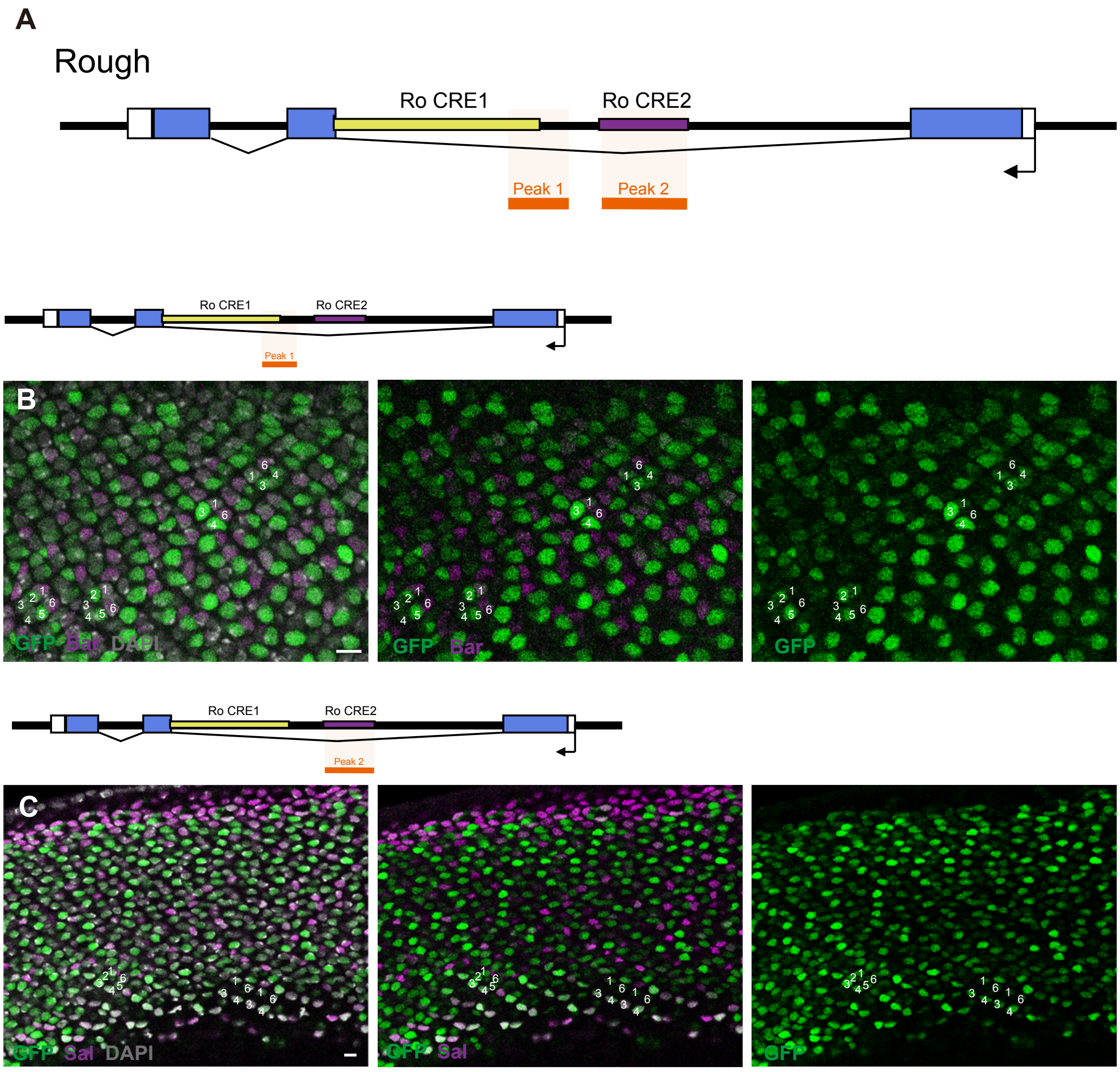

A
Rough
Ro CRE1
Ro CRE2
Peak 1
Peak 2
B
GFP Bar DAPI
GFP Bar
GFP
C
GFP Sal DAPI
GFP Sal
GFP

Figure S6. Rough CRE testing in *Drosophila*.

(**A**) Overview of the *Rough* gene in the *Drosophila* genome. Two Rough CREs were tested in intron 2, chosen partly based on single cell ATAC Seq data from (*53*), with peaks shown in light orange. (**B**) Ro CRE 1 driving expression of GFP (green) in the *Drosophila* 3rd instar larval eye disc. Expression was variable in PRs 1-6. (**C**) Ro CRE 2 driving expression of GFP (green) in outer PRs 1-6, with Sal (magenta) labeling R3/4. Scale bar, 5µm.

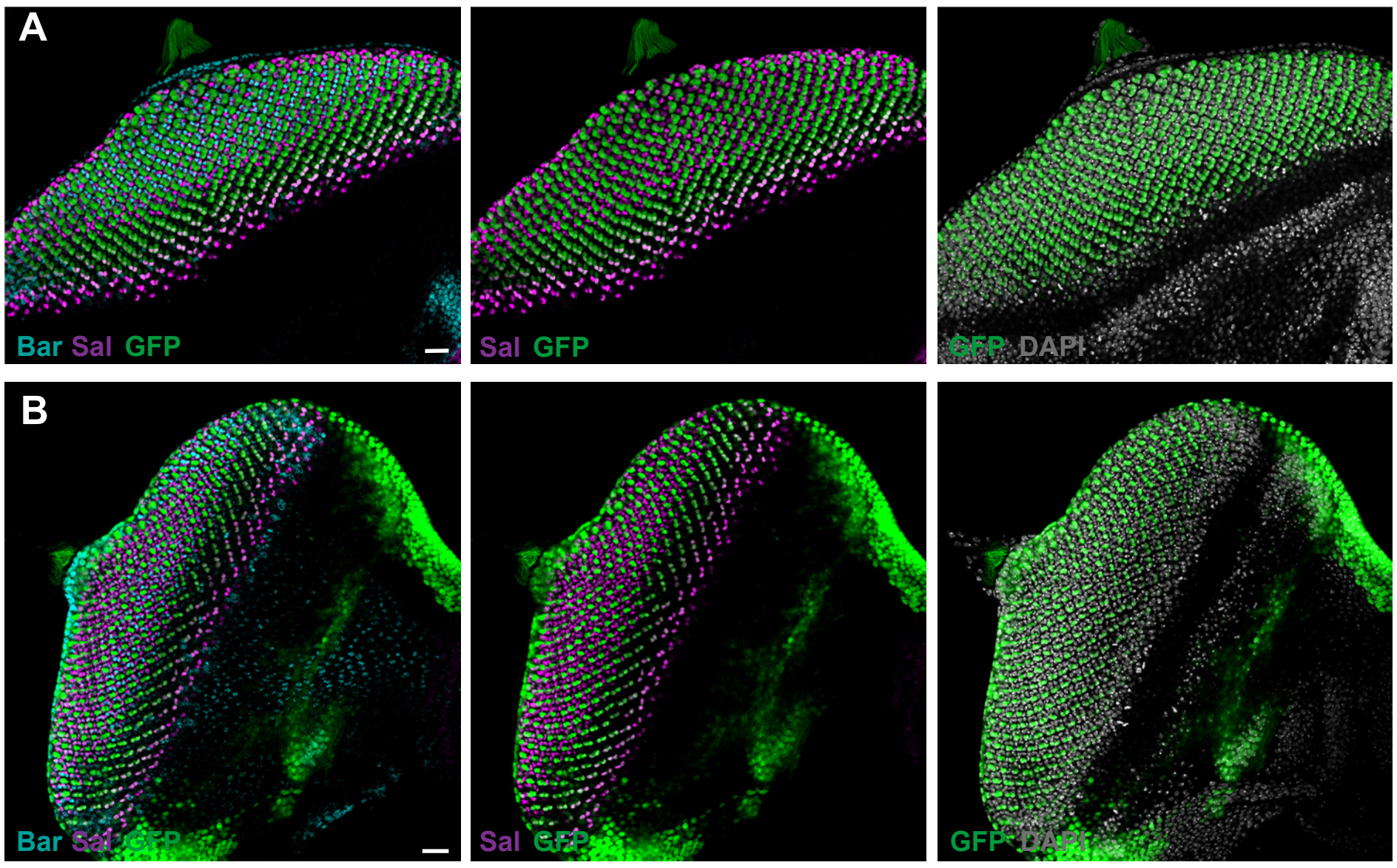
A
Bar Sal GFP
Sal GFP
GFP DAPI
B
Bar Sal GFP
Sal GFP
GFP DAPI

Figure S7. Two R3/4 CRE candidates in *Drosophila*.

(**A**) R55F01 from the CG14509 locus drive reporter expression in R2/5 and R3/4. GFP (green) labels R2/5 and R3/4. Sal (magenta) labels R3/4. Bar (cyan) labels R1/6. (**B**) R86F02 from the Klumpfuss (Klu) locus drive reporter expression in R3/4. GFP (green) labels R3/4. Sal (magenta) labels R3/4. Bar (cyan) labels R1/6. All nuclei are labeled with DAPI (gray). Scale bar, 20μm.

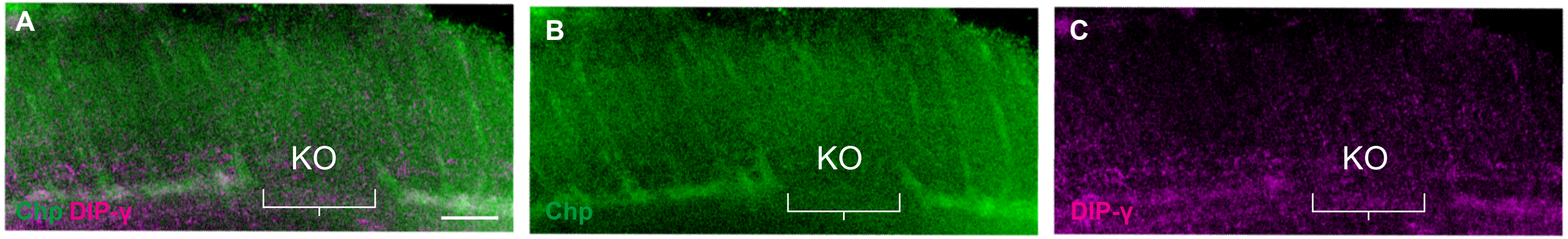
A
KO
Chp DIP-γ
B
KO
Chp
C
KO
DIP-γ

Figure S8. The effect of R7 loss on Dm8 retention in butterfly brains.
(**A** to **C**) CRISPR-induced mosaic knockout of *Lz* results in the loss of R7 axons and DIPγ expression in the *Vanessa* medulla. The *Lz* KO region (indicated by bracket) lacks Chp (green, B) and DIPγ (magenta, C), which are visible in adjacent WT regions. Scale bar, 10μm.

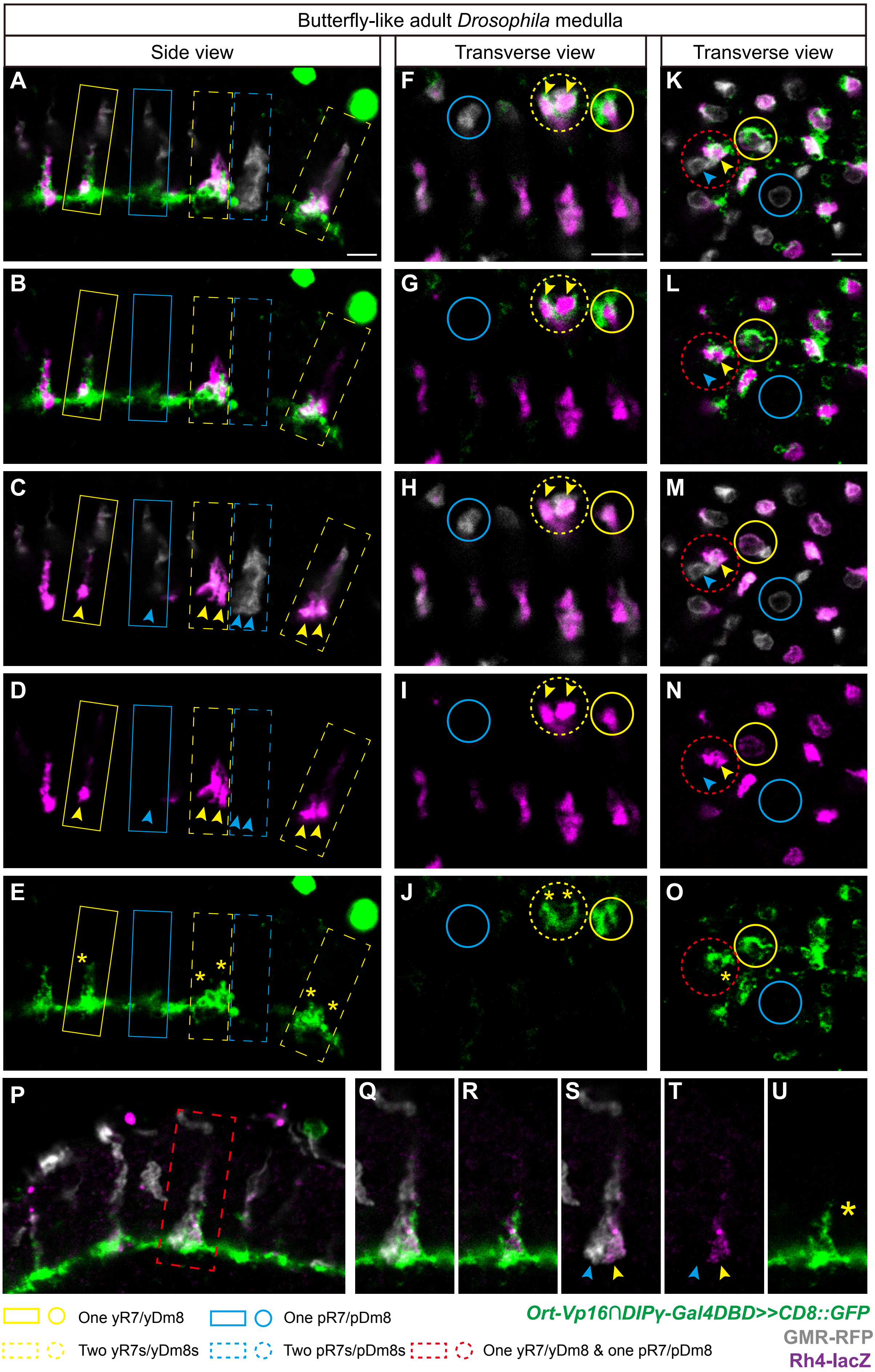

Butterfly-like adult Drosophila medulla
Side view
Transverse view
Transverse view
A
B
C
D
E
F
G
H
I
J
K
L
M
N
O
P
Q
R
S
T
U
One yR7/yDm8
One pR7/pDm8
Two yR7s/yDm8s
Two pR7s/pDm8s
One yR7/yDm8 & one pR7/pDm8
Ort-Vp16∩DIPγ-Gal4DBD>>CD8::GFP
GMR-RFP
Rh4-lacZ

Figure S9. Specific connectivity between subtype R7 and Dm8 in butterfly-like adult *Drosophila* brain.

(**A** to **E**) Side view of the adult medulla in *sev-lz*, *sev-Su(H)enR* flies. Compared with WT columns containing one yR7 (yellow solid rectangle) and one pR7 (blue solid rectangle), yellow dashed rectangles highlight two yR7 axons (labelled by βGalactosidase, magenta) contacting two DIPγ-positive yDm8 neurons (green) within the same home column. Two yellow arrowheads indicate the two yR7 axons and two yellow asterisks mark the two DIPγ-positive yDm8 neurons in the same home column. Blue dashed rectangle highlights two pR7 axons (labelled by GMR-RFP, gray). Two blue arrowheads indicate the two pR7 axons and no DIPγ-positive yDm8 neurons are observed in the same home column. (**F** to **O**) Transverse view of the adult medulla in *sev-lz*, *sev-Su(H)enR* flies. Compared with WT columns containing one yR7 (yellow solid circle) and one pR7 (blue solid circle), yellow dashed circles in (F to J) highlight two yR7 terminals labelled by βGalactosidase (magenta, yellow arrowheads) that connect to two DIPγ-positive yDm8 neurons (green, yellow asterisks) within a single column. Red dashed circles in (K to O) highlight two R7 terminals, one yR7 labelled by βGalactosidase (magenta, yellow arrowhead) and one pR7 labelled by GMR-RFP (gray, blue arrowhead) within a single home column, in which one DIPγ-positive yDm8 neuron (green) connects to the yR7 terminal and no DIPγ-positive yDm8 neuron contacts the pR7 terminal. (**P** to **U**) Side view of the adult medulla in *sev-lz*, *sev-Su(H)enR* flies. The red dashed rectangle in (P) highlights two R7 axons, one yR7 axon labelled by βGalactosidase (magenta, yellow arrowhead) and one pR7 axon labelled by GMR-RFP (gray, blue arrowhead) within a single home column, in which one DIPγ-positive yDm8 neuron (green, yellow asterisk) connects to the yR7 axon and no DIPγ-positive yDm8 neuron contacts the pR7 axon. Scale bar, 5μm.

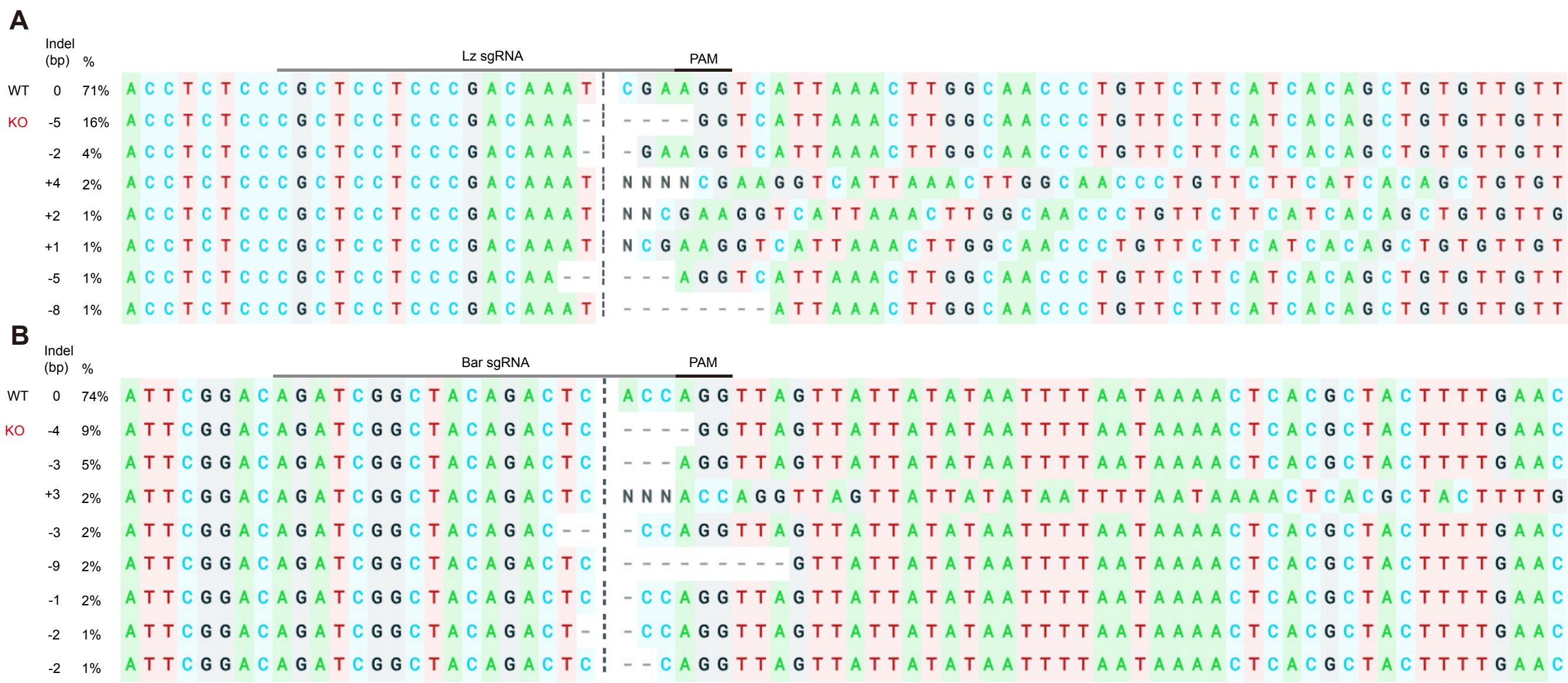
A
Indel (bp)
%
Lz sgRNA
PAM
WT 0 71%
KO -5 16%
-2 4%
+4 2%
+2 1%
+1 1%
-5 1%
-8 1%
B
Indel (bp)
%
Bar sgRNA
PAM
WT 0 74%
KO -4 9%
-3 5%
+3 2%
-3 2%
-9 2%
-1 2%
-2 1%
-2 1%

Figure S10. Genotyping validation of CRISPR-induced mosaic mutations.
(**A** and **B**) Sanger sequencing showing indels at the *Bar* (A) and *Lz* (B) target sites in *Vanessa* using Synthego ICE analysis. Predicted cut sites are indicated by dotted lines.

Table S1: Six of the 61 lines from the Janelia FlyLight collection that were evaluated as potential R3/4 enhancers. potential R3/4 enhancers.

Number	Stock no.	Gene name
R65A05	*39329*	*abl*
R86F06	*46824*	*klu*
R86F02	*46823*	*klu*
R55F01	*39124*	*CG14509*
R22E03	*61641*	*pkcdelta*
R24C05	*49313*	*argos*

Table S2 Primers and sgRNA guides used in this study

1. CRISPR sgRNA	
Gene name	Sequences
Lozenge	TCTAGCACGTCGCCCAGGGC
	CGCTCCTCCCGACAAATCGA
Bar	TATACTCGACGCTGCGCCGA
	AGATCGGCTACAGACTCACC
2. CRISPR screening primers	
Gene name	Sequences
Lozenge	CGGGTACGTGCTTCTAtcttatg
	CTTCAAGGTAGTGGCCCTGG
Bar	GAAGGACGGCGATGGTTAAT
	CGGGTACGTGCTTCTAtcttatg

Table S3 Antibody Generation

Protein name	Sequence of antigen used. Added His tags indicated in **bold**.
VcBar (XP_046976157.1)	MTVQRDERDARAPRTRFMITDILDAAPRDLSAHRDSDSDRSATDSPGVKDDS<br>DDVSSKSCGGDASGLAKKQRKARTAFTDHQLQTLEKSFERQKYLSVQDRME<br>LAAKLGLTDTQVKTWYQNRRTKWKRQTAVGLELLAEAGNYAAFQRLYGGYW<br>AGVPAYPAQPTPTADLYYRQAAATAAAAASASANTLQKPLPYRLYPGAPLGGV<br>PPLGLGLPGPSAHLGSLGAPGLGALGYYAQARRTPSPDVDPGSPAPPPRSPR<br>EPSIEQRSDDEDDDETIHV
VcRo (XP_046967584.2)	MDKEIYPEKSKDTSIQTPRPSSPRKFFARIYGHLDQPKTKDKEQTQGSESDSCSD<br>VEIGDNEGRDSLSASPLWPPPPLPVCPRPILLPHQQLAFGAGLAAFLARRRRKES<br>RPRRQRTTFSAHQTLRLELEYARGEYVARARRCELASALSLSETQVKIWFQNRRA<br>KDKRIEKAHLDQQYRQLAAASGFFQPIFNSPYGPPPCPCLPVHSPPNLEK**HHHHH**<br>**H**
VcLz (XP_046969678.1)	MHLPYESRGRRRREMAELEPWWLQSIIDETLGEHPDLVRTGSPDYMCSMLPQH<br>WRSNKTLPGGFKVVALGDVLDGTQVTVRAGNDENCSAELRNNTAVMKNRVAKFN<br>DLRFVGRSGRGKSFSLTITVSTTPPQVATYQKAIKVTVDGPREPRSKTSTNASTHQ<br>IRTIGFQRPFISGSSMALREMEYKSSARSLRSLSHEEREYKTNANLPTEENTGNIL<br>GASEWNTGYPSSASVYPSYGPLQPAYYKPDPTIHIPAVLPEIPLGHTSDYSGFQSG<br>STGFVKGSPSGASGTLTDLNTPAMTPQRYDPNYYNSWPANSYNYQYNNINNNPA<br>CLQSHTPYINPNPQMILPNLTYSTVNQNQIHVHLHSSSDKYNLEQYMPSEIKINDID<br>GGISITTDLPGVGEPSGLVQTCESADDVKNGLYGSGSQEVWRPY**HHHHHH**
VcChaoptin (XM_047111186.1)	MYGLDNTLWELELKQDRLTSVPSRALRYLQKLRLLDLTGNEITEITGDNWLGLENS<br>LQTLILSDNSIATLPLDAFSGLLILETLDLHGNHLSVIDSGVFRDGMSRLSKLLLGDN<br>QLTLIPYEELSPLRQLRHLDLSNNLIKQVPPAHDLNGVKLSLDYLKLDQNNIKILLPG<br>SFKYFNILNTTSLNGNPIFTIREDAFRNAKIKTLSLRDCGVTELSPASFAGLENSLQN<br>LDLSENNLTMISKFMLNKLDSLRFLNLRENKVDTNLLATNNPSEYSTTPSVNNFQY<br>KLFYLDISGSSSLEMSLQDVRRMRSLRYLAVSKLIRRSITSEDFLEFGVELEDLKIIG<br>STINRIEASAFQHVRTIKTLDLSENNIDFIDPFAFAELHSLTTLKMANGLADSVKILPF<br>EPLKALIELEYLDLSNNKLKNVPDTSFHFMYKLKTLNLQDNLIDQFSKGTLQSDIHR<br>ELESVCLSLNQLQRIDQHTFVNLRELQEILIEDNLIETVYRRSFTSLDNLKVIRLRGN<br>IITEISEESFQNLPALKELDISFNQLETFKFSIFDQVGSATALKVNVSYNRIISLTDSNA<br>PSFFTSNFYPPPKAQRLVSEDPSPLRIERGLGTVSVNIRVLDFSHNNISYIAPYYFR<br>HADLTLSELYLSYNLIRNVTREVFGSMLMLQYLDLSHNQIFHMEYDCFKKVKNLQII<br>DLSHNHLIELPVEVFHDMQALTSVDLSDNNIKNLADNLIISPSLERLDLSDNDLSRIP<br>TNSLSPAAAINLVELDLSGNTIPAVAIADLVQ**HHHHHH**
VcDIP-γ (XP_046966872.1)	M**HHHHHH**LKDSQTTTKKFSEEPAFERPIGNHTFFLGREAVLGCAV<br>TNLGKHKVGWLRAEDQTVLTMHERAVLGSRYAVSLDAPRTWQLRIRPLRAEDRG<br>CYMCQINTQPTMTWQIGCIDVFVPPDIVSDDTSGDVSVQELENATLTCKATGHPP<br>PKITWRREDHEPILLKKPSSRDFDKVESYVGSSMPLWRVDRRQMGAFLCIASNDV<br>PPAVSKRITLNVNFAPTVKVPNQLLGAPLGTDVKLKCYVEAYPNTINYWIKNRGEM<br>LLDGPKYTIREEKTSYKVSMWLTIRQFSKSDIGTYNCVSTNSLGKSEGTLRLYEIKL<br>NSYSEDFSNQISVAGGLTEAAKGNSEDKFRIERTLLIMLFCIVFMLT

Table S4: Sequences used to design HCR probes

Gene	Sequences
Vc_RunxB (XM_047113547.1)	MHLSSEVSSTTNGLGHEAPSSSYGNALPSQPLTAELLAERTLEGLLADH<br>PGELVKTGCPHVVCTVLPPHWRSNKTLPVAFKVVALGDVGDGTLVTVR<br>AGNDENCSAELRNCTAVMKNQVAKFNDLRFVGRSGRGKSFTLTIMLATS<br>PPQVATYQKAIKVTVDGPREPRSKTRHHGFHPFHFGPRFAPDPLMGSL<br>PFKLSGIAHQLAGLPGGEWGALARHYPPPLLPTHAHFTPHVLPPAHAQH<br>HTVPPPPHETDLASDNPNTMHPHSTTSPVNSRPSSPQDDDDISVTASS<br>SPPPDDRPGAFTSVTRPRKPLQPLPAPSSLFHSALAAQLFLNSPLLPTP<br>PSWLYSQLYGGYDWWLRPPPQQDDSNSSPDREEEAGSTASSSTKKRP<br>ASPEWTDQGVRTRSKSLITSERRPVDVWRPY
Vc_Dpr11 (XM_047110961.1)	MCACAWRLHAFTAALVLAAHLIKVLSCGETGGGGGTRARRYVGLYTGP<br>YFDPSAPNNITAQLGTHAYLPCKVRQLSNKSVSWIRRRDAHILTVDRFTF<br>IADERFQAFLVEATDTWTLQVKYVQARDAGVYECQVGTEPKMSHFVQL<br>NVVVPKIEIVGESDLYVKAGSTVSLKCVITQALEEPAYIFWYHNDERVLNY<br>DRSLVEIRMERLAPDTTIGNLIIYNPRREDSGNYSCSPSNLDSASVVLHV<br>LSGEQPAAMQHGNGAAPTRARALLLSAAAALAAPRRSRVLALLALALILP<br>LVAVPRRRLTKIASAARRLARTVDPVKYRVLRVRDACPRTSPIRYKLLLSL<br>EPEGSDDGASSFNILSIISRRVHRWDLSTSGDRLRVIIYFRRRLRSGAAS<br>ARRRRGVGAASARRRRGVGAQARPAFVKLYDEQ
Vc_Cut (XM_047108033.1)	MHPTGAASLPAAPEAEVQAMHSMDWLFKKERIYLLAQFWQQVIKSTTQ<br>PINTRATLAEKEVTTLKEQLATTSPTPLQATVPPKTNGSHIESTRDQATET<br>RIPERFSPDIKEEKRRSPDIDEDIEQKIEMAATARSNSNSSRSSPVVNQS<br>SSLENELAAKEKEIAQLVEDVRRLQASLSALQEAHAQQLQRLEERLDEK<br>KQHIARLEARLDTQRDYDEIKREISMLRFSELGPTERPHSHTLPKDIFGS<br>QNHLLRSMDLGPNERVERKEALRSPAAPAHRDSSAERERSTERRDTG<br>GDEWPSTPPPLNNNTTHHNNNGPVPLPLPPPSPFRFEEHRPYRFAED<br>MGPLPPGALVGRLGDSLIPKGDPMEARLQEMLRYNMDKYANSNLDTLH<br>ISRRVRELLSVHNIGQRLFAKYVLGLSQGTVSELLSKPKPWDKLTEKGR<br>DSYRKMHAWACDEAAIMLLKSLIPKKVGTKDGSAGTGFGRPEGEGDER<br>LAHMLNEASHLMKTPTGQPNNDDSRSNEDSSSPRTQCPSPFSKDSSQ<br>NRRLKKYENDDIPQEKVVRIYQEELAKIMTRRVEDMRHNREGFPGVFPP<br>FFSGGMPPHMERPPEDIRMALEAYHRELAKIQPGGNIPNLHNMPGMPP<br>FPNLLALQQQALQAQNQHMNGSGAVQDLSLPKDKNTKINGLTDSDKDR<br>SMDAEEAIRHAGSAFSLVRPKLEPGQQSTGSSASSPLGNAILPPAITPND<br>DFSNSAAASPLQRMASITNSLISQPPNPPHHAPPQRSMKAVLPPITQQQ<br>FDLFNNLNTEEIVKRVKEALSQYSISQRLFGESVLGLSQGSVSDLLARPK<br>PWHMLTQKGREPFIRMKMFLEDDNAVHKLVASQYKIAPEKLMRTGNYS<br>GAPPCPPNMNKPMPPTQKMISDATSLLSKMQQEQLLSSGHLGHLGQPT<br>PLLLTPPGFPPHHAVTLPPQHHDNNNKERKPPPPPQPHHQPPVMRGLH<br>QHMSPSVYEMAALTQDLDTQTITTKIKEALLANNIGQKIFGEAVLGLSQG<br>SVSELLSKPKPWHMLSIKGREPFIRMQLWLSDAHNIDRLQALKNERREA<br>NKRRRSSGPGQDNSSDTSSNDTSEFYHSSSPGPTSGVPSAKKQRVLF

	SEEQKEALRLAFALDPYPNTPTIEFLAAELGLSTRTITNWFHNHRMRLKQHAPHGLPAEPPARDQTAAPFDPVQFRLLLNQRLLELQKERMGLAGVPLPYPPYFAANSNFAALIGRGLLPPEERVKDPTSGLDLSMPLKREPDGDDFEDDDVESNLGSDDSLDEESKNEPKAASTPASRSNRRKPAAPQWVNPDWQDEKPRNPDEVIINGVCVMRSDDFRREAEETVRVEPSPVPREPSPAASPASRASRASPASLASHARSLARTPDVLPEDKIKTEAEDDRWDY
Vc_Side_VII (XP_046971678.1)	MIEIIMPLALCRFIASAAILGVAVATTARNAPLTQVVAVAGEPVYLPCDVATQDEDDAVLLVLWYREDLGTPIYSVDAREKDFGVAERWSDESVFASRAYFLPERRPAELGVDRVRATDQGIYRCRVDFKQAQTRNSRVNLTVIVPPNKMVITDDKGDIKQAIVGPYVEGDTFTLKCDVSGGRPRPWVKWFRNELETDTPATTLQGTSVRGVLRVGPLTRADVRATLTCRASNHLRAHPIETTLTLDMNFPPLNVHILGSNQPLSASRRYDLLCQSSGSRPPASITWWKNGHRINNAKETLSTDGNTTTSTVSIQLNKADAGAKLACRASNPQMPSIPAMEDDWLLDIQYVPETVVRLGTNLDPSNIREGSDVYFDCIIKAHPYVYKVEWRHNGKTLHHNVGQGIIISNQSLVLQGVGRKTAGNYTCVGFNAEGDGESKAFSLNVLYAPTCRSSQQRVHGVAKQERAHITCHVDANPPEVSFRWTFNNTANSNEVSDTYVSRSGTSSTVTYTPHTEMDYGTLLCWAHNRIGKQRVPCVYHIIAAGRPDQVHNCTVVNASLTSFGVRCSEGFNGGMPQSFLLEVREIVTQEIVANVSSAVPRFTAVGLVPGRTYAAAVLAYNAKGRGDPYPLRASTLRPPEKHHVHDKSLDLPRTAFQMSGAMSAAVGGGGVLMVVLVLFMIAVRQRCAKRKPRSAPPPSQPASPDKLREKDDSESDDRNPDIIPSDTDHNMQMDYYRQQQVSTISPPSSRARGSIAGVRGGLPAYCALRTAPPSDSTPPSGIPPPAGFASGSCTLPRNAGTANERCIPMQHLRTLPRAHPHPHPHPHVHAHAHPHPPRDTPL
Vc_Svp (XP_046966929.1)	HTRAPPRSHPATATGIITHGALVYGGVGGPGLCPPRASDLPPLELGFRGSWREDELLPSTPASQAASTQSGSSANDKGQNVECVVCGDKSSGKHYGQFTCEGCKSFFKRSVRRNLTYSCRGNRNCPIDQHHRNQCQYCRLRKCLKMGMRREAVQRGRVPPTQPAGLALPGQFALANGDPAAGLNSHPYLSSYISLLLRAEPYPTSRYGQCVQPTNVMGIDNICELAARLLFSAVEWARNIPFFPELQVTDQVALLRLVWSELFVLNASQCSMPLHVAPLLAAAGLHASPMAADRVVAFMDHIRIFQEQVEKLKALHVDSAEYSCLKAIVLFTTDACGLSDVPHIESLQEKSQCALEEYCRSQYPNQPTRFGKLLLRLPSLRTVSSQVIEQLFFVRLVGKTPIETLIRDMLLSGSSFSWPYMATM
Vc_Runt (XM_047113541.1)	MHLPHASPPTPTMADVYTHIHEYYRQSHGDLVQTGSPAVLCSALPGHWRSNKSLPVAFKVVALDDVQDGTLVTIKAGNDENVMAEMRNCTAVMKNQVAKFNDLRFVGRSGRGKSFSLTITISTFPSQVATYTKAIKVTVDGPREPRTKQNYGYGHPGAFNPFLLNPGWLDAAYLNYAWADYFRPPQLRDQTLVKGGSTPITTPPVALPGGELFPFPPAMASLPSGGLIPPPGAFLPPNGLLPFPPHPAELALKTLPPELTLKNGINPYDALRHLQSNVSSMDTSSARLSPTSSRQSGSPRSIVNASPRSKADSKSEVNSTHEATISDESDEEQIEVVKSAFHPTRPANVELQEMKQVQAADSTVSDRPRMRNELKATSQRTTRVLSTSPTSTKITNGSISTHKSVWRPY

Data S1. Seurat FindAllMarkers output for each cell type cluster.

REFERENCES

1. M. A. Tosches, Developmental and genetic mechanisms of neural circuit evolution. *Dev. Biol.* **431**, 16–25 (2017).

2. N. Konstantinides, C. Desplan, Neuronal circuit evolution: From development to structure and adaptive significance. *Cold Spring Harb. Perspect. Biol.* **17**, a041493 (2025).

3. G. H. Jacobs, G. A. Williams, H. Cahill, J. Nathans, Emergence of novel color vision in mice engineered to express a human cone photopigment. *Science* **315**, 1723–1725 (2007).

4. J. Neitz, J. Carroll, Y. Yamauchi, M. Neitz, D. R. Williams, Color perception is mediated by a pastic neural mechanism that is adjustable in adults. *Neuron* **35**, 783–792 (2002).

5. V. Hamburger, R. Levi-Montalcini, Proliferation, differentiation and degeneration in the spinal ganglia of the chick embryo under normal and experimental conditions. *J. Exp. Zool.* **111**, 457–501 (1949).

6. R. W. Oppenheim, Cell death during development of the nervous system. *Annu. Rev. Neurosci.* **14**, 453–501 (1991).

7. C. J. Axelrod, S. P. Gordon, B. A. Carlson, Integrating neuroplasticity and evolution. *Curr. Biol.* **33**, R288–R293 (2023).

8. M. F. Wernet, E. O. Mazzoni, A. Çelik, D. M. Duncan, I. Duncan, C. Desplan, Stochastic spineless expression creates the retinal mosaic for colour vision. *Nature* **440**, 174–180 (2006).

9. J. Ainsworth, Y. Zhao, K. Gao, N. M. Gravina, Z. H. Goldberg, C. Pinkerton, S. A. Rifkin, A. M. Ernst, H. G. Garcia, M. W. Perry, Cell fate ratios are encoded by transcriptional dynamics in the *Drosophila* retina. *Curr. Biol.* **35**, 2946–2959.e5 (2025).

10. J. Rister, C. Desplan, D. Vasiliauskas, Establishing and maintaining gene expression patterns: Insights from sensory receptor patterning. *Development* **140**, 493–503 (2013).

11. N. Franceschini, K. Kirschfeld, B. Minke, Fluorescence of photoreceptor cells observed in vivo. *Science* **213**, 1264–1267 (1981).

12. B. S. Wells, D. Pistillo, E. Barnhart, C. Desplan, Parallel Activin and BMP signaling coordinates R7/R8 photoreceptor subtype pairing in the stochastic *Drosophila* retina. *Elife* **6**, e25301 (2017).

13. M. Friedrich, E. J. Wood, M. Wu, Developmental evolution of the insect retina: Insights from standardized numbering of homologous photoreceptors. *J. Exp. Zool. B Mol. Dev. Evol.* **316**, 484–499 (2011).

14. M. Perry, M. Kinoshita, G. Saldi, L. Huo, K. Arikawa, C. Desplan, Molecular logic behind the three-way stochastic choices that expand butterfly colour vision. *Nature* **535**, 280–284 (2016).

15. K. Gao, A. Donati, J. Ainsworth, D. Wu, E. R. Terner, M. W. Perry, Deep conservation complemented by novelty and innovation in the insect eye ground plan. *Proc. Natl. Acad. Sci. U.S.A.* **122**, (2025).

16. K. Arikawa, S. Mizuno, M. Kinoshita, D. G. Stavenga, Coexpression of two visual pigments in a photoreceptor causes an abnormally broad spectral sensitivity in the eye of the butterfly *Papilio xuthus*. *J. Neurosci.* **23**, 4527–4532 (2003).

17. J.-Y. Roignant, J. E. Treisman, Pattern formation in the *Drosophila* eye disc. *Int. J. Dev. Biol.* **53**, 795–804 (2009).

18. A. Tomlinson, Y. E. Mavromatakis, G. Struhl, Three distinct roles for Notch in *Drosophila* R7 photoreceptor specification. *PLoS Biol.* **9**, e1001132 (2011).

19. A. Tomlinson, G. Struhl, Delta/Notch and Boss/Sevenless signals act combinatorially to specify the *Drosophila* R7 photoreceptor. *Mol. Cell* **7**, 487–495 (2001).

20. K. K. Bollepogu Raja, K. Yeung, Y.-K. Shim, Y. Li, R. Chen, G. Mardon, A single cell genomics atlas of the *Drosophila* larval eye reveals distinct photoreceptor developmental timelines. *Nat. Commun.* **14**, 7205 (2023).

21. A. C. Miller, E. A. Urban, E. L. Lyons, T. G. Herman, R. J. Johnston Jr., Interdependent regulation of stereotyped and stochastic photoreceptor fates in the fly eye. *Dev. Biol.* **471**, 89–96 (2021).

22. A. Tomlinson, D. F. Ready, Cell fate in the *Drosophila* ommatidium. *Dev. Biol.* **123**, 264–275 (1987).

23. A. Tomlinson, Cellular interactions in the developing *Drosophila* eye. *Development* **104**, 183–193 (1988).

24. G. V. Flores, A. Daga, H. R. Kalhor, U. Banerjee, Lozenge is expressed in pluripotent precursor cells and patterns multiple cell types in the *Drosophila* eye through the control of cell-specific transcription factors. *Development* **125**, 3681–3687 (1998).

25. J. R. Crew, P. Batterham, J. A. Pollock, Developing compound eye in *lozenge* mutants of *Drosophila*: Lozenge expression in the R7 equivalence group. *Dev. Genes Evol.* **206**, 481–493 (1997).

26. P. Batterham, J. R. Crew, A. M. Sokac, J. R. Andrews, G. M. F. Pasquini, A. G. Davies, R. F. Stocker, J. A. Pollock, Genetic analysis of the *Lozenge* gene complex in *Drosophila melanogaster*: Adult visual system phenotypes. *J. Neurogenet.* **10**, 193–220 (1996).

27. B. E. Kimmel, U. Heberlein, G. M. Rubin, The homeo domain protein rough is expressed in a subset of cells in the developing *Drosophila* eye where it can specify photoreceptor cell subtype. *Genes Dev.* **4**, 712–727 (1990).

28. S. Higashijima, T. Kojima, T. Michiue, S. Ishimaru, Y. Emori, K. Saigo, Dual Bar homeo box genes of *Drosophila* required in two photoreceptor cells, R1 and R6, and primary pigment cells for normal eye development. *Genes Dev.* **6**, 50–60 (1992).

29. P. M. Domingos, S. Brown, R. Barrio, K. Ratnakumar, B. J. Frankfort, G. Mardon, H. Steller, B. Mollereau, Regulation of R7 and R8 differentiation by the spalt genes. *Dev. Biol.* **273**, 121–133 (2004).

30. Y. E. Mavromatakis, A. Tomlinson, Switching cell fates in the developing *Drosophila* eye. *Development* **140**, 4353–4361 (2013).

31. E. Agi, M. Langen, S. J. Altschuler, L. F. Wu, T. Zimmermann, P. R. Hiesinger, The evolution and development of neural superposition. *J. Neurogenet.* **28**, 216–232 (2014).

32. S. Gao, S. Takemura, C.-Y. Ting, S. Huang, Z. Lu, H. Luan, J. Rister, A. S. Thum, M. Yang, S.-T. Hong, J. W. Wang, W. F. Odenwald, B. H. White, I. A. Meinertzhagen, C.-H. Lee, The neural substrate of spectral preference in *Drosophila*. *Neuron* **60**, 328–342 (2008).

33. K. P. Menon, V. Kulkarni, S. Takemura, M. Anaya, K. Zinn, Interactions between Dpr11 and DIP-γ control selection of amacrine neurons in *Drosophila* color vision circuits. *Elife* **8**, e48935 (2019).

34. M. Courgeon, C. Desplan, Coordination between stochastic and deterministic specification in the *Drosophila* visual system. *Science* **366**, eaay6727 (2019).

35. R. A. Carrillo, E. Özkan, K. P. Menon, S. Nagarkar-Jaiswal, P.-T. Lee, M. Jeon, M. E. Birnbaum, H. J. Bellen, K. C. Garcia, K. Zinn, Control of synaptic connectivity by a network of *Drosophila* IgSF cell surface proteins. *Cell* **163**, 1770–1782 (2015).

36. C. Anderson, I. Reiss, C. Zhou, A. Cho, H. Siddiqi, B. Mormann, C. M. Avelis, P. Deford, A. Bergland, E. Roberts, J. Taylor, D. Vasiliauskas, R. J. Johnston, Natural variation in stochastic photoreceptor specification and color preference in *Drosophila*. *Elife* **6**, e29593 (2017).

37. A. Nern, B. D. Pfeiffer, G. M. Rubin, Optimized tools for multicolor stochastic labeling reveal diverse stereotyped cell arrangements in the fly visual system. *Proc. Natl. Acad. Sci. U.S.A.* **112**, E2967–E2976 (2015).

38. S. Takemura, A. Bharioke, Z. Lu, A. Nern, S. Vitaladevuni, P. K. Rivlin, W. T. Katz, D. J. Olbris, S. M. Plaza, P. Winston, T. Zhao, J. A. Horne, R. D. Fetter, S. Takemura, K. Blazek, L.-A. Chang, O. Ogundeyi, M. A. Saunders, V. Shapiro, C. Sigmund, G. M. Rubin, L. K. Scheffer, I. A. Meinertzhagen, D. B. Chklovskii, A visual motion detection circuit suggested by *Drosophila* connectomics. *Nature* **500**, 175–181 (2013).

39. A. Nern, F. Loesche, S. Takemura, L. E. Burnett, M. Dreher, E. Gruntman, J. Hoeller, G. B. Huang, M. Januszewski, N. C. Klapoetke, S. Koskela, K. D. Longden, Z. Lu, S. Preibisch, W. Qiu, E. M. Rogers, P. Seenivasan, A. Zhao, J. Bogovic, B. S. Canino, J. Clements, M. Cook, S. Finley-May, M. A. Flynn, I. Hameed, A. M. C. Fragniere, K. J. Hayworth, G. P. Hopkins, P. M. Hubbard, W. T. Katz, J. Kovalyak, S. A. Lauchie, M. Leonard, A. Lohff, C. A. Maldonado, C. Mooney, N. Okeoma, D. J. Olbris, C. Ordish, T. Paterson, E. M. Phillips, T. Pietzsch, J. R. Salinas, P. K. Rivlin, P. Schlegel, A. L. Scott, L. A. Scuderi, S. Takemura, I. Talebi, A. Thomson, E. T. Trautman, L. Umayam, C. Walsh, J. J. Walsh, C. S. Xu, E. A. Yakal, T. Yang, T. Zhao, J. Funke, R. George, H. F. Hess, G. S. X. E. Jefferis, C. Knecht, W. Korff, S. M. Plaza, S. Romani, S. Saalfeld, L. K. Scheffer, S. Berg, G. M. Rubin, M. B. Reiser, Connectome-driven neural inventory of a complete visual system. *Nature* **641**, 1225–1237 (2025).

40. M. F. Wernet, M. W. Perry, C. Desplan, The evolutionary diversity of insect retinal mosaics: Common design principles and emerging molecular logic. *Trends Genet.* **31**, 316–328 (2015).

41. K. J. McCulloch, F. Yuan, Y. Zhen, M. L. Aardema, G. Smith, J. Llorente-Bousquets, P. Andolfatto, A. D. Briscoe, Sexual dimorphism and retinal mosaic diversification following the evolution of a violet receptor in butterflies. *Mol. Biol. Evol.* **34**, 2271–2284 (2017).

42. X. Qiu, K. Arikawa, The photoreceptor localization confirms the spectral heterogeneity of ommatidia in the male small white butterfly, *Pieris rapae crucivora*. *J. Comp. Physiol. A Neuroethol. Sens. Neural Behav. Physiol.* **189**, 81–88 (2003).

43. K. Mancuso, W. W. Hauswirth, Q. Li, T. B. Connor, J. A. Kuchenbecker, M. C. Mauck, J. Neitz, M. Neitz, Gene therapy for red–green colour blindness in adult primates. *Nature* **461**, 784–787 (2009).

44. J. S. Wu, L. Luo, A protocol for mosaic analysis with a repressible cell marker (MARCM) in *Drosophila*. *Nat. Protoc.* **1**, 2583–2589 (2006).

45. G. Sancer, E. Kind, J. Uhlhorn, J. Volkmann, J. Hammacher, T. Pham, H. Plazaola-Sasieta, M. F. Wernet, Cellular and synaptic adaptations of neural circuits processing skylight polarization in the fly. *J. Comp. Physiol. A Neuroethol. Sens. Neural Behav. Physiol.* **206**, 233–246 (2020).

46. M. T. D. Cooper, S. J. Bray, Frizzled regulation of Notch signalling polarizes cell fate in the *Drosophila* eye. *Nature* **397**, 526–530 (1999).

47. A. Tomlinson, G. Struhl, Decoding vectorial information from a gradient: Sequential roles of the receptors Frizzled and Notch in establishing planar polarity in the *Drosophila* eye. *Development* **126**, 5725–5738 (1999).

48. U. Heberlein, G. M. Rubin, Structural and functional comparisons of the *Drosophila virilis* and *Drosophila melanogaster* rough genes. *Proc. Natl. Acad. Sci. U.S.A.* **87**, 5916–5920 (1990).

49. U. Heberlein, A. Penton, S. Falsafi, D. Hackett, G. M. Rubin, The C-terminus of the homeodomain is required for functional specificity of the *Drosophila* rough gene. *Mech. Dev.* **48**, 35–49 (1994).

50. B. D. Pfeiffer, A. Jenett, A. S. Hammonds, T.-T. B. Ngo, S. Misra, C. Murphy, A. Scully, J. W. Carlson, K. H. Wan, T. R. Laverty, C. Mungall, R. Svirskas, J. T. Kadonaga, C. Q. Doe, M. B. Eisen, S. E. Celniker, G. M. Rubin, Tools for neuroanatomy and neurogenetics in *Drosophila*. *Proc. Natl. Acad. Sci. U.S.A.* **105**, 9715–9720 (2008).

51. J. F. de Celis, R. Barrio, F. C. Kafatos, Regulation of the *spalt/spalt-related* gene complex and its function during sensory organ development in the *Drosophila* thorax. *Development* **126**, 2653–2662 (1999).

52. E. Kuehn, D. S. Clausen, R. W. Null, B. M. Metzger, A. D. Willis, B. D. Özpolat, Segment number threshold determines juvenile onset of germline cluster expansion in *Platynereis dumerilii*. *J. Exp. Zool. B Mol. Dev. Evol.* **338**, 225–240 (2022).

53. C. Bravo González-Blas, X. Quan, R. Duran-Romaña, I. I. Taskiran, D. Koldere, K. Davie, V. Christiaens, S. Makhzami, G. Hulselmans, M. de Waegeneer, D. Mauduit, S. Poovathingal, S. Aibar, S. Aerts, Identification of genomic enhancers through spatial integration of single-cell transcriptomics and epigenomics. *Mol. Syst. Biol.* **16**, e9438 (2020).
